# Infection with Possible Precursor of Avian Influenza A(H7N9) Virus in a Child, China, 2013

**DOI:** 10.3201/eid2008.140325

**Published:** 2014-08

**Authors:** Lili Ren, Xuelian Yu, Baihui Zhao, Fan Wu, Qi Jin, Xi Zhang, Jianwei Wang

**Affiliations:** Ministry of Health Key Laboratory for Systems Biology of Pathogens, Beijing, China (L. Ren, Q. Jin, J. Wang);; Institute of Pathogen Biology, Beijing (L. Ren, Q. Jin, J. Wang);; Shanghai Municipal Center for Disease Control and Prevention, Shanghai, China (X. Yu, B. Zhao, F. Wu, X. Zhang);; Shanghai Jiao Tong University, Shanghai (B. Zhao)

**Keywords:** avian influenza virus, viruses, A(H7N9), phylogenetics, evolution, adaption, genome sequence, pediatric, zoonoses, respiratory infections, fomite, China

## Abstract

During the early stage of the avian influenza A(H7N9) epidemic in China in March 2013, a strain of the virus was identified in a 4-year-old boy with mild influenza symptoms. Phylogenetic analysis indicated that this strain, which has similarity to avian subtype H9N2 viruses, may represent a precursor of more-evolved H7N9 subtypes co-circulating among humans.

Influenza A(H7N9) virus infected >400 persons in China during March 2013–April 2014 (*1*–*3*) in China. Although this virus does not appear to be readily transmitted from person to person, its identification in a wide geographic area of China and discovery of amino acid changes associated with mammalian adaption of the virus have caused increased concerns for a pandemic (*1*,*2*). 

The origin and evolution of the H7N9 subtype have been discussed intensively based on the results of phylogenetic analysis of the available sequences (*1*,*4*–*8*). The hemagglutinin (HA) and neuraminidase (NA) genes of the H7N9 subtype that circulated among humans during 2013 were possibly introduced from wild birds that carried differing subtype H9N2 strains and then reassorted in domestic birds such as chickens (*4*–*6*). A/brambling/Beijing/16/2012(H9N2) (BJ16)–like virus and/or other related avian virus H9N2 strains are proposed to be the sources of the internal genes of the 2013 H7N9 subtypes (*5*,*7*). However, the precise source and evolution route of strains in human H7N9 subtypes have not been well established (*4*–*6*). Intermediate or precursor strains are extrapolated to exist at the interface between avian and human H7N9 subtypes (*5*,*6*), but such strains have not been identified.

Here we report the identification of a distinct strain, A/Shanghai/JS01/2013(H7N9) (SH/JS01), which was detected in a patient with mild influenza symptoms in Shanghai during March 2013, during the very early stage of the influenza A(H7N9) epidemic. Phylogenetic analysis indicates that this strain may represent an earlier precursor of the more evolved H7N9 subtypes co-circulating at low levels at the time of isolation in March 2013 thus providing insight into the evolution of H7N9 subtypes.

## The Study

A mild case of influenza A(H7N9) virus infection was identified in a 4-year-old boy in a rural area of Jinshan District, Shanghai, reported on March 31. The patient had been exposed to poultry. His signs and symptoms included acute fever (maximum 39° axillary), cough, nasal drainage, and tonsillitis. A diagnosis of upper respiratory tract infection was made, and the child recovered after 5 days of antiviral drug therapy (*9*,*10*). Nasal swab specimens were positive for influenza A(H7N9) virus by using real-time RT-PCR (*11*), as recommended by the World Health Organization. Although his family members, unrelated workers, and chickens he may have had contact with were tested, none tested positive for influenza virus.

The whole genome sequence of the SH/JS01strain was amplified from the nasal swab specimen by using RT-PCR (primer sequences available upon request). Strict controls were used during PCR amplification; results were confirmed by another laboratory to exclude contamination with laboratory strains. We constructed maximum likelihood trees of each gene segment sequence using the general time-reversible model implemented in MEGA 5.1 (*12*), and estimated divergence time using the Bayesian Markov chain Monte Carlo method implemented in BEAST (v1.6.1) (*13*). We compared all known strains of the 2013 H7N9 subtype and other referenced influenza virus sequences deposited in GISAID (http://platform.gisaid.org/epi3/frontend#57f951) and GenBank (Table 1 and Technical Appendix Figures 1–8).

**Table 1:** The GenBank accession numbers, gene clade, and estimated divergent time of the sequences for A/Shanghai/JS01/2013*

Gene segment	GenBank accession no.	Gene clade†	Estimated time of divergence‡
PB2	KF609508	Minor	Jul 2010
PB1	KF609509	Major	Jun 2002
PA	KF609510	Minor	Mar 2012
HA	KF609511	ND	Oct 2005
NP	KF609512	Major	Jan 2001
NA	KF609513	ND	Sep 2010
M	KF609514	Minor	May 2011
NS	KF609515	ND	Oct 1996

*PB, polymerase basic subunit; PA, RNA polymerase acidic subunit; HA, hemagglutinin; NP, nucleoprotein; NA, neuraminidase; M, matrix gene; NS, nonstructural gene. †Defined according to reference (*5*); ND, not divided: HA, NA, and NS genes were not divided into clades. ‡Calculations based on sequences in Technical Appendix

The critical mutations in the SH/JS01 strain associated with virulence and mammalian adaption were compared to 3 prevalent H7N9 subtype reference strains: A/Shanghai/1/2013 (SH/1), A/Shanghai/2/2013 (SH/2), and A/Anhui/1/2013 (AH/1). In the HA gene of SH/JS01, the only mammalian adapting substitution observed was 138A (H3 numbering); amino acid residues involved in receptor-binding specificity showed avian-like signatures, including 186G and 226Q, which were similar to SH/1 but distinctive from SH/2 and AH/1. In the internal genes of SH/JS01, we observed some human-like and mammalian-adapting signatures, including 89V in polymerase basic subunit (PB)2, 368V in PB1, 356R in the RNA polymerase acidic subunit, 42S in nonstructural gene 1, and 30D and 215A in matrix gene 1; however, some hallmark changes involved in mammalian adaptation still showed avian signatures, including 627E and 701D in PB2 and 100V and 409S in the RNA polymerase acidic subunit (Table 2). Most strikingly, SH/JS01 retained aa 69–73 (N9 numbering) in the stalk region of the NA gene. In contrast, deletion of aa 69–73, which is considered to occur when viruses adapt to terrestrial birds, prevailed in all the known 2013 H7N9 subtype isolates (*14*) (Table 2, and Figure). These findings indicate that SH/JS01 is genetically distinct from all the known human influenza A(H7N9) strains and carries more avian influenza-like signatures.

**Table 2:** Analysis on critical mammalian-adapting amino acid substitutions in H7N9 virus strains.*†

Gene	Site	SH/JS01	SH/1	SH/2	AH/1	Known mutations	Relationship to mammalian adaption
HA	138	**A**	S	**A**	**A**	S138A	Mammalian host adaption
	186	G	G	**V**	**V**	G186V	Unknown
	226	Q	Q	**L**	**L**	Q226L	Unknown
	228	G	G	G	G	G228S	Unknown
NA	292	R	**K**	R	R	R292K	Osteltamivir and zanamivir resistance
	69–73 deletion	No	**Yes**	**Yes**	**Yes**	Not applicable	Deletion of 69–73 Increased virulence in mice
PB2	63	I	I	I	I	I63T	Co-mediate with PB1 677M, to reduce pathogenicity of H5N1 viruses
	89	**V**	**V**	**V**	**V**	L89V	Enhanced polymerase activity and increased virulence in mice
	471	T	T	T	T	T471M	Viral replication, virulence, and pathogenicity
	591	Q	Q	Q	Q	Q591K	Adapt in mammals that compensates for the lack of PB2–627K
	627	E	**K**	**K**	**K**	E627K	Enhanced polymerase activity and increased virulence in mice
	701	D	D	D	D	D701N	Enhanced transmission in guinea pigs
PB1	99	H	H	H	H	H99Y	Results in transmissible of H5 virus among ferrets
	353	K	K	K	K	K353R	Determine viral replication, virulence, and pathogenicity
	368	**V**	I	**V**	**V**	I368V	Results in transmissible of H5 virus among ferrets
	566	T	T	T	T	T566A	Determine viral replication, virulence, and pathogenicity
	677	T	T	T	T	T677M	Co-mediate with PB2 I63T to reduce pathogenicity of H5N1 viruses
PA	100	V	**A**	**A**	**A**	V100A	Related to human adaption
	356	**R**	**R**	**R**	**R**	356R	Related to human adaption
	409	S	**N**	**N**	**N**	S409N	Enhances transmission in mammals
M1	30	**D**	**D**	**D**	**D**	N30D	Increased virulence in mice
	215	**A**	**A**	**A**	**A**	T215A	Increased virulence in mice
M2	31	**N**	**N**	**N**	**N**	S31N	Reduced susceptibility to amantadine and rimantadine
NS1	42	**S**	**S**	**S**	**S**	P42S	Increased virulence in mice

*HA, hemagglutinin; NA, neuraminidase; SH/JS01, A/Shanghai/JS01/2013(H7N9); SH/1, A/Shanghai/1/2013 (H7N9); SH/2, A/Shanghai/2/2013 (H7N9); AH/1, A/Anhui/1/2013 (H7N9); PB, RNA polymerase basic subunit; PA, RNA polymerase acidic subunit; M, matrix gene; NS, nonstructural gene. †Boldface text indicates the mutant amino acids sites related to mammalian host adaption and increased virulence.

**Figure:**
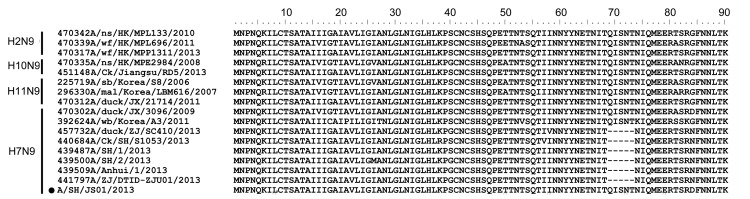
Amino acid sequence alignment of the neuraminidase (NA) stalk region. The dark circle indicates the sequence characterized in this study. The abbreviations of the sequence names are as follows: ns, northern shoveler; wf, wild waterfowl; Ck, Chicken; Sb, shorebird; mal, mallard; wb, wild bird; HK, Hong Kong; JX, Jiangxi; ZJ, Zhejiang; SH, Shanghai.

Phylogenetic analysis and divergence time estimation showed that the SH/JS01 HA gene diverged in October 2005 and was closely related to SH/1; nucleotide similarity was 99.7% (online Technical Appendix Figure 1). However, the NA gene, which is most closely related to A/northern shoveler/Hong Kong/MPL133/2010(H2N9) and A/duck/Jiangxi/21714/2011(H11N9) with nucleotide similarity of 99% and 99.3%, respectively, are estimated to have diverged in September 2010, earlier than that of known strains of the 2013 H7N9 subtype (estimated to have occurred in January 2011) (Table 1; Technical Appendix Figure 2).

On the basis of internal genes, 2013 H7N9 viruses have been divided into minor (m) and major (M) clades in the phylogenetic trees, and first 9, then 27 genotypes (*5*,*6*,*8*). Our data showed that SH/JS01 belongs to the m-PB2|PA|M or G3 genotype (Table 1; Technical Appendix Figures 3–8); its NS gene and that of A/Chicken/Dawang/1/2011(H9N2) (DW1), shared the highest nt identity (99.4%). The SH/JS01 NS gene had an estimated divergence time as early as October 1996 (Table 1; Technical Appendix Figure 3). The closest relatives of the SH/JS01 M gene were poultry H9N2 strains A/chicken/Jiangsu/CZ1/2012 and A/chicken/Jiangsu/NTTZ/2013 (nt identity 99.7%); BJ16 (98.6%) was not closely related. The divergence time of SH/JS01 M gene is estimated as May 2011, which is earlier than most 2013 H7N9 isolates (Table 1; Technical Appendix Figure 4). The PB2 and PA genes of SH/JS01 were positioned in minor clades and showed higher identities to A/chicken/Jiangsu/MYJMF/2012(H9N2) (99.2% and 99.3%, respectively) than to BJ16 (97.1% and 98%) (Table 1; Technical Appendix Figures 5, 6). In contrast, the PB1 and NP genes of SH/JS01 belong to the major lineage (Technical Appendix Figures 7, 8). Collectively, these data indicate that, except for PB1 and NP, the internal genes of SH/JS01 are more closely related to those in poultry H9N2 viruses identified before 2013 than to BJ16, which was an H9N2 virus considered to be the donor of most of internal genes of the 2013 H7N9 virus.

## Conclusions

SH/JS01, a distinct H7N9 virus strain identified in 2013 during the early stage of the influenza A(H7N9) epidemic in China, provided information to define the evolution of the H7N9 subtype. Although identified in an infected human, SH/JS01 has more avian-prone properties and fewer mammalian-adapting mutations than other known human 2013 H7N9 subtypes. SH/JS01 has a waterfowl-like NA gene characterized by the absence of a deletion in the NA stalk and most of its internal genes are more closely related to avian H9N2 subtype strains isolated during the 2011–2012 influenza season than to other recently emerged strains of the H7N9 subtype. Molecular clock analysis further predicted an earlier divergence time in most genes of SH/JS01. These findings indicate that SH/JS01 might be a precursor strain of the H7N9 virus that co-circulated with more evolved viruses, although we cannot exclude that SH/JS01 may have been generated independently from the other H7N9 strains by reassortment of waterfowl strains with avian H9N2 strains and then transmitted directly to a human.

The sequences of SH/JS01 contained more avian-like signatures than those of other H7N9 isolates from humans; this underscores the potential of these viruses to infect humans. The phenotypic characteristics of SH/JS01, which might describe its zoonotic potential, remain to be investigated. 

It is unclear whether other SH/JS01–like viruses are still circulating in poultry in China and if so, what the potential is for their evolution and ability to infect humans. Intensive influenza surveillance and additional influenza A virus genome sequences isolated from poultry and from humans with severe and mild manifestations of infection are needed to clarify the population dynamics of H7N9 viruses.

Technical Appendix Phylogenetic analysis of avian influenza viruses, 1988–2013
