# Supplementary material for: Infection with Possible Precursor of Avian Influenza A(H7N9) Virus in a Child, China, 2013
Source: Emerg Infect Dis. 2014 Aug;20(8):1362–5. doi: 10.3201/eid2008.140325 (PMC4111171; doi:10.3201/eid2008.140325)
Supplement: Technical Appendix — Phylogenetic analysis of avian influenza viruses, 1988–2013 [file 14-0325-Techapp-s1.pdf]

DOI: http://dx.doi.org/10.3201/eid2008.140325

# Infection with Possible Precursor of Avian Influenza A(H7N9) Virus in a Child, China, 2013

## Technical Appendix: Phylogenetic analysis of avian influenza viruses, 1988–2013

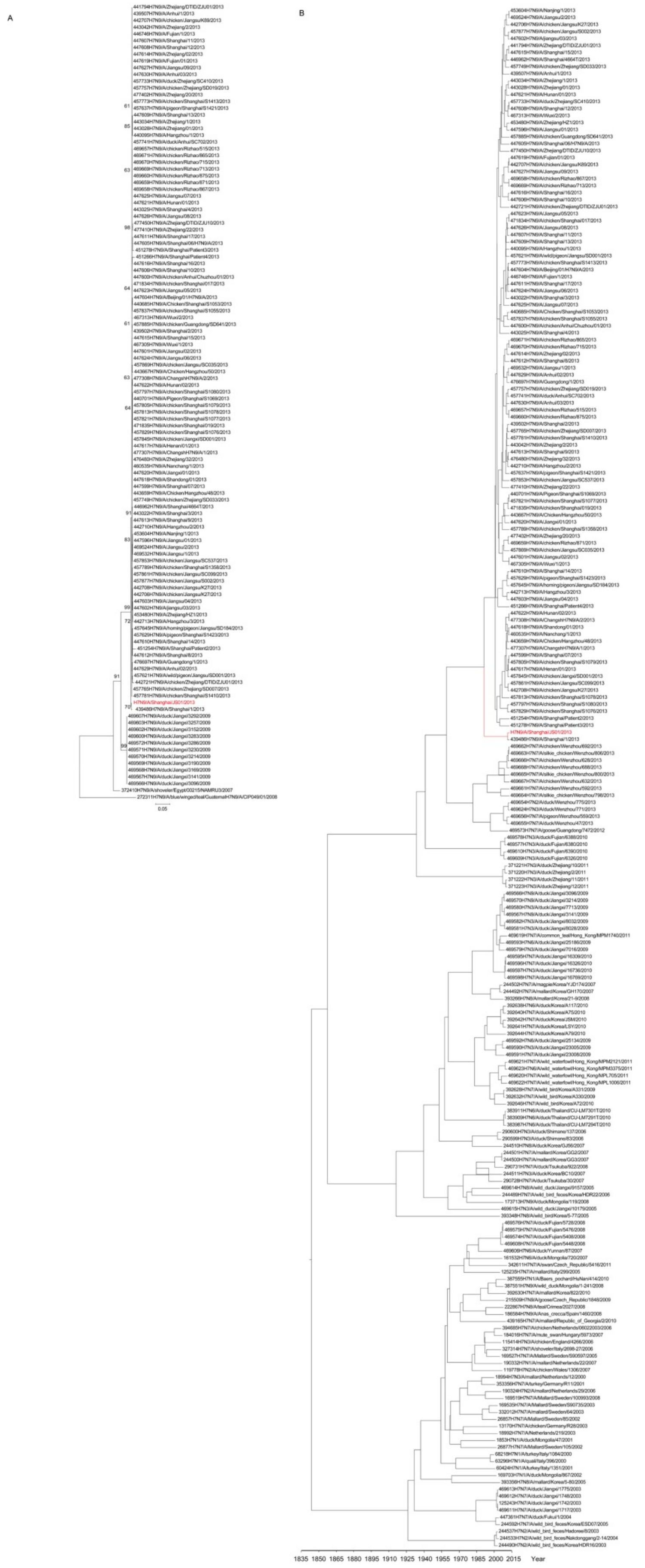

Technical Appendix Figure 1. Phylogenies of hemagglutinin (HA) gene of A/Shanghai/JS01/2013(H7N9). A) We constructed the maximum likelihood tree (n = 118) using MEGA5.1 with the general time reversible model and with 1,000 bootstrap pseudo-replicates. The A/Shanghai/JS01/2013(H7N9) strain isolated in this study is highlighted in red. B) We accomplished molecular clock analysis (n = 220) using Bayesian Markov chain Monte Carlo method implemented in Bayesian evolutionary analysis by sampling trees (http://dx.doi.org/10.1186/1471-2148-7-214). The timescale bar is provided at the bottom. The A/Shanghai/JS01/2013(H7N9) strain is highlighted in red.

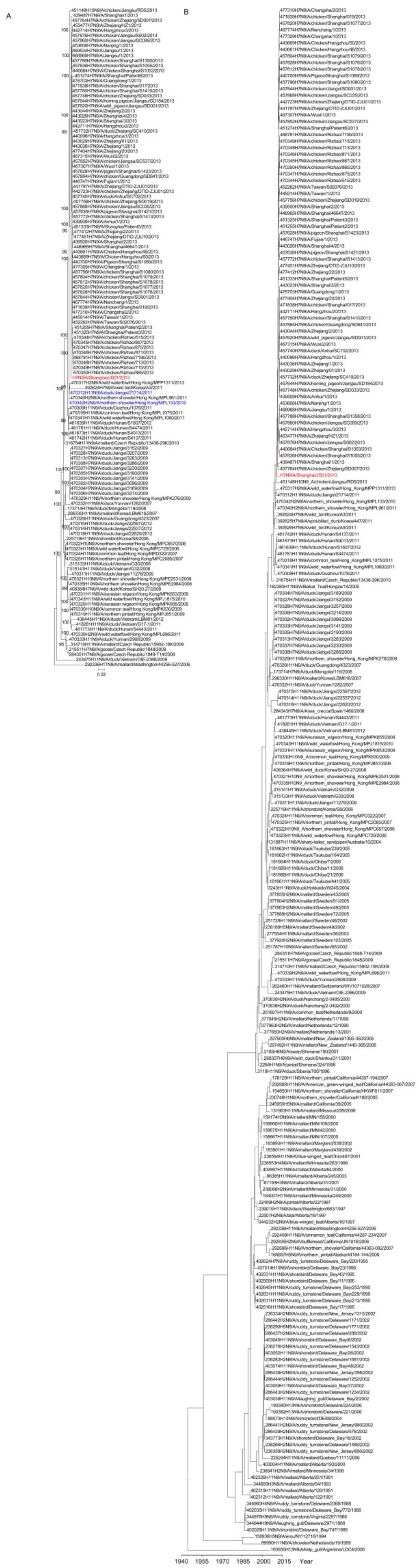

Technical Appendix Figure 2. Phylogenies of neuraminidase (NA) gene. A) Maximum likelihood tree (n = 131). The close relatives of chicken strains are highlighted in blue. B) Time-scaled tree (n = 236). Annotation as in Technical Appendix Figure 1.

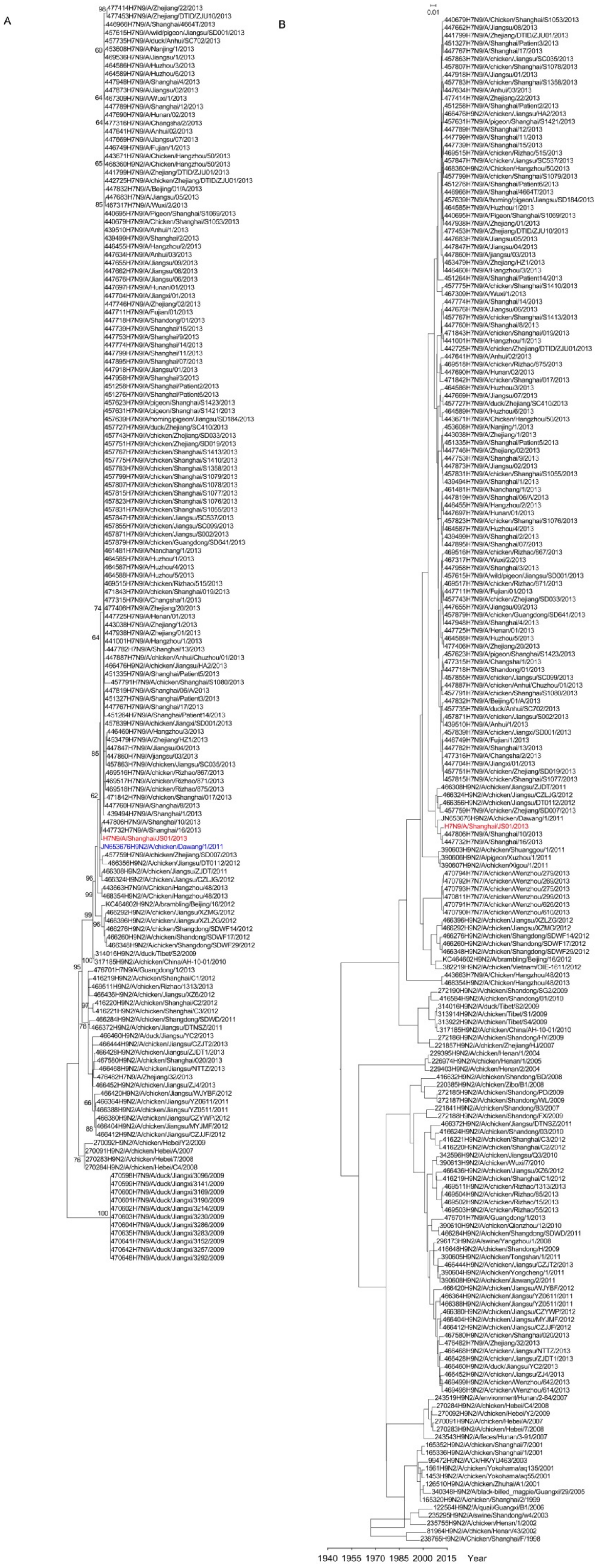


Technical Appendix Figure 3. Phylogenies of non-structural gene (NS). A) Maximum likelihood tree (n = 153). The close relatives of chicken strains are highlighted in blue. B) Time-scaled tree (n = 195). Annotation as in Technical Appendix Figure 1.

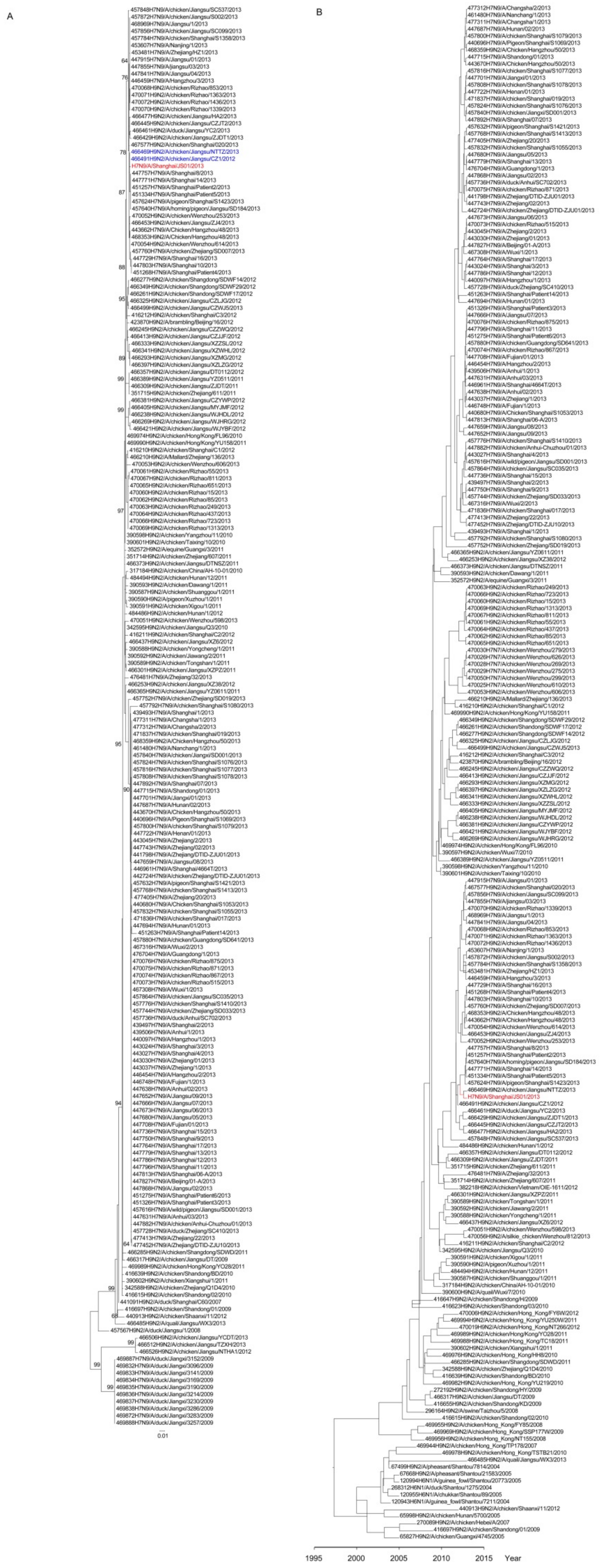


Technical Appendix Figure 4. Phylogenies of matrix gene (M). A) Maximum likelihood tree (n = 200). The close relatives of chicken strains are highlighted in blue. B) Time-scaled tree (n = 220). Annotation as in Technical Appendix Figure 1.

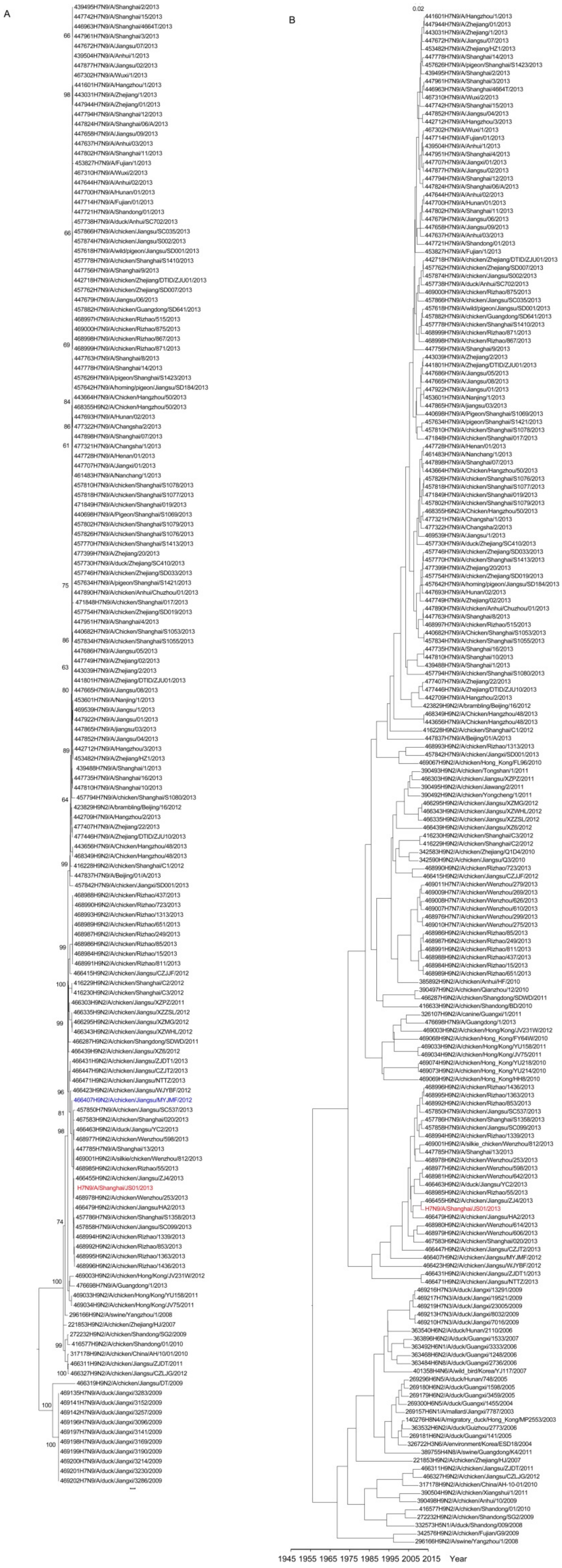


Technical Appendix Figure 5. Phylogenies of RNA polymerase basic subunit 2 (PB2). A) Maximum likelihood tree (n = 152). The close relatives of chicken strains are highlighted in blue. B) Time-scaled tree (n = 189). Annotation as in Technical Appendix Figure 1.

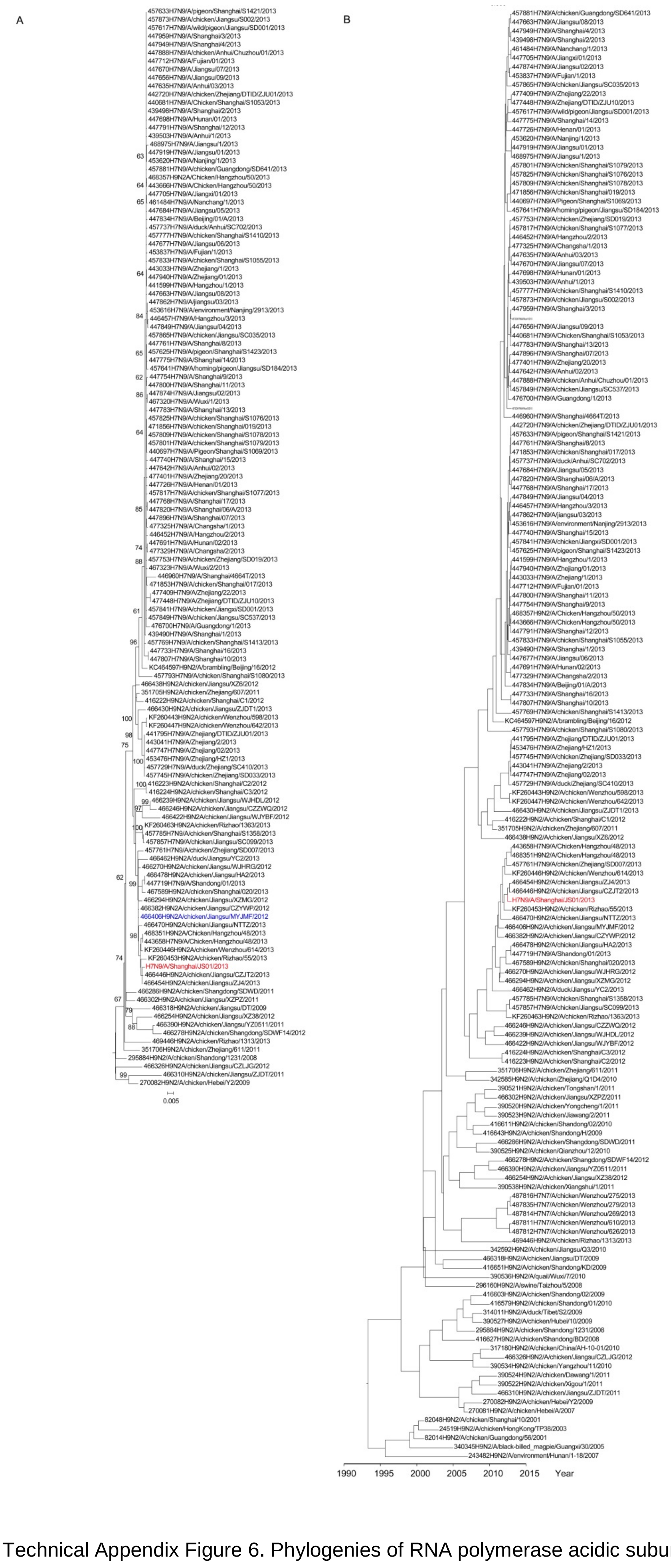


Technical Appendix Figure 6. Phylogenies of RNA polymerase acidic subunit (PA). A) Maximum likelihood tree (n = 130). The close relatives of chicken strains are highlighted in blue. B) Time-scaled tree (n = 162). Annotation as in Figure 1.

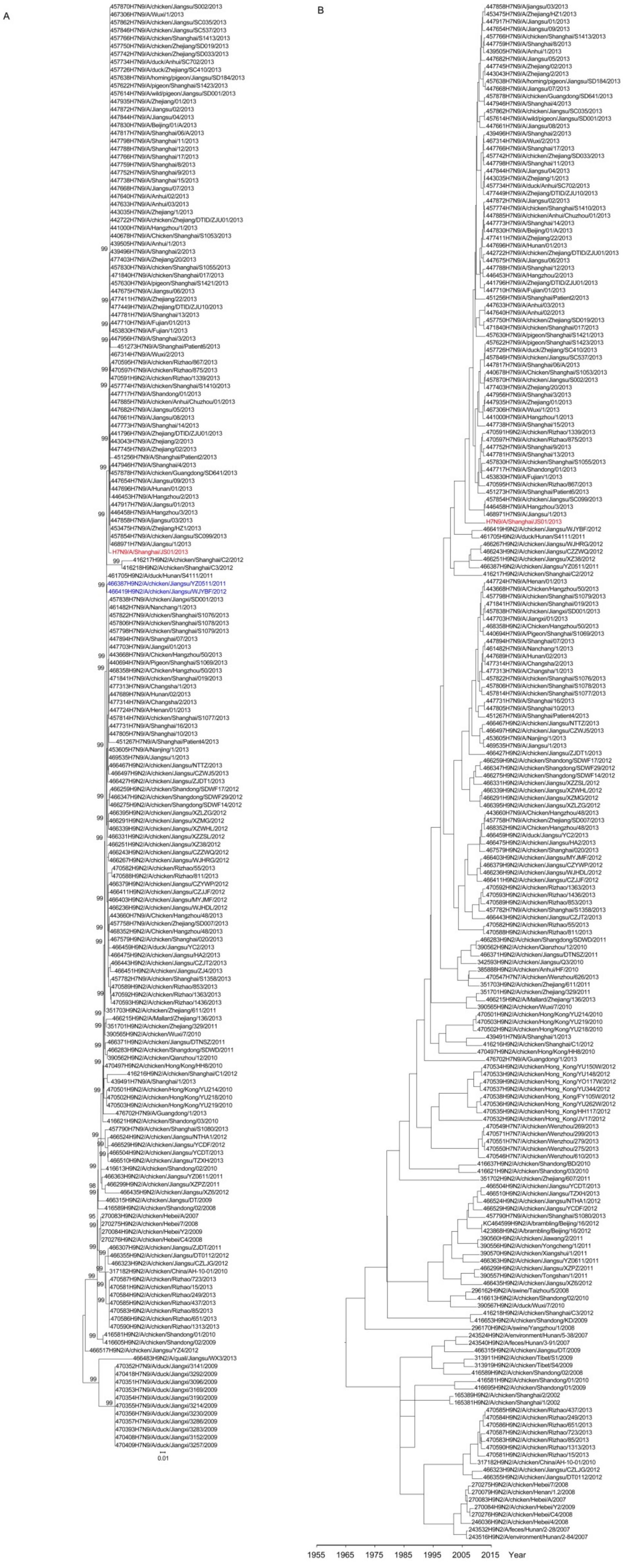


Technical Appendix Figure 7. Phylogenies of nucleocapsid protein (NP) gene. A) Maximum likelihood tree (n = 184). The close relatives of chicken strains are highlighted in blue. B) Time-scaled tree (n = 206). Annotation as in Technical Appendix Figure 1.

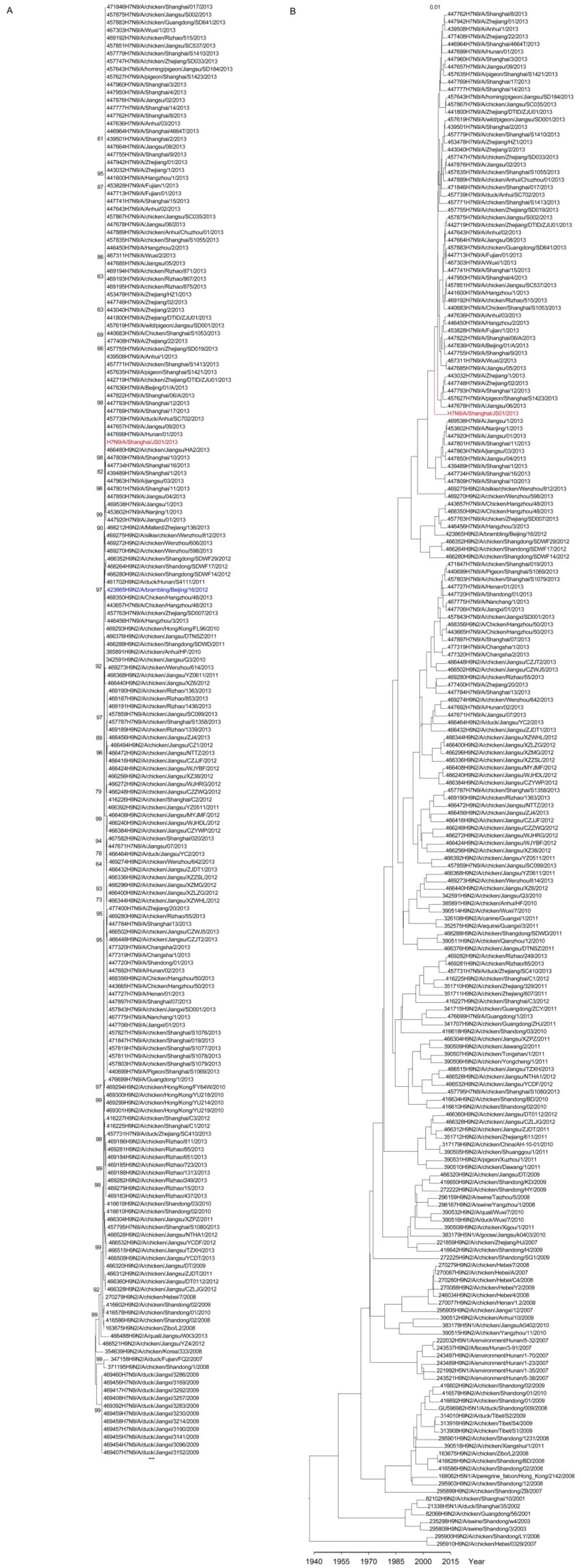


Technical Appendix Figure 8. Phylogenies of RNA polymerase basic subunit 1(PB1). A) Maximum likelihood tree (n = 187). The close relatives of chicken strains are highlighted in blue. B) Time-scaled tree (n = 204). Annotation as in Technical Appendix Figure 1.
